# Necrotizing Pneumonia Caused by Panton-Valentine Leucocidin-Producing *Staphylococcus aureus* Originating from a Bartholin's Abscess

**DOI:** 10.1155/2008/491401

**Published:** 2008-07-29

**Authors:** N. Jung, C. Lehmann, M. Hellmann, H. Seifert, M. M. Valter, M. Hallek, G. Fätkenheuer, M. Kochanek

**Affiliations:** ^1^Department I of Internal Medicine, University of Cologne, 50935 Cologne, Germany; ^2^Institute for Medical Microbiology, Immunology and Hygiene, University of Cologne, 50935 Cologne, Germany; ^3^Clinic for Gynecology, University of Cologne, 50935 Cologne, Germany

## Abstract

*Background*. Panton-Valentine leukocidin (PVL-)producing *Staphylococcus aureus* is emerging as a serious problem worldwide. There has been an increase in the incidence of necrotizing lung infections in otherwise healthy young people with a very high mortality associated with these strains. Sporadic severe infectious complications after incision of Bartholin's abcesses have been described but involvement of *S. aureus* is rare. *Case report*. We present a 23-year-old apparently healthy female patient without any typical predisposing findings who developed severe sepsis with necrotizing pneumonia and multiple abscesses following incision of a Bartholin's abscess. Methicillin-sensitive *S. aureus* harbouring Panton-Valentine leucocidin genes were cultured from the abscess fluid, multiple blood cultures and a postoperative wound swab. Aggressive antibiotic therapy with flucloxacillin, rifampicin and clindamycin, drainage and intensive supportive care lead finally to recovery. *Conclusions*. *S. aureus*, in particular PVL-positive strains, should be considered when a young, immunocompetent person develops a fulminant necrotizing pneumonia. Minor infections—such as Bartholin's abscess—can precede this life-threating syndrome. Bactericidal antistaphylococcal antibiotics are recommended for treatment, and surgical procedures may become necessary.

Panton-Valentine leucocidin (PVL) is a pore-forming cytotoxin of *Staphylococcus aureus* inducing leukocyte lysis. It has been associated with diverse clinical syndromes, including primary and secondary skin infections such as furunculosis and abscesses and deep seated infections such as necrotizing pneumonia. Its role and regulation are still under investigation [1]. *S. aureus* has the capacity to produce a wide array of virulence factors which are responsible for divergent clinical syndromes [2]. In the US, the most common circulating community-associated methicillin-resistant *S. aureus* strain USA 300 contains PVL, but most infections are skin and soft tissue infections, including boils, and are not life threatening [3].

Necrotizing pneumonia due to PVL-positive *S. aureus* is usually severe and often fatal, involves primarily young and healthy patients, and carries a mortality rate up to 75% despite intensive medical treatment [4].

To our knowledge, we present the first case of necrotizing pneumonia following incision of a Bartholin's abscess. In October 2006, a 23-year-old woman with no systemic signs of infection had a surgical incision of a Bartholin's abscess (3 × 5 × 3 cm) without the installation of a drainage. The patient was discharged without antimicrobial therapy. The abscess fluid was submitted for culture.

24 hours later, the woman developed fever and her state of health worsened continuously, so she was admitted to a local hospital 4 days after surgery. Community-acquired pneumonia was diagnosed and a macrolide was administered. One day later, the patient became critically ill and was transferred to our tertiary care hospital and immediately admitted to the intensive care unit.

She presented with an influenza-like illness with severe muscle pain in all extremities, spiking fever up to 40°C, dry cough but no sore throat. She showed ortho- and tachypnoea with a respiratory rate of 30 breaths/minute, oxygen saturation was 96% with the supply of 31/minute of oxygen but no need for intubation. Her blood pressure was 100/60 mmHg and her heart rate 140/minute. Physical examination revealed crackles in the inferior and middle parts of both lungs, a diffuse tenderness of the abdomen, and severe pain in the muscles. Auscultation of the heart was unremarkable. Vaginal examination revealed a postoperative wound without any signs of infection. She denied ever having used illicit drugs.

Admission laboratory data revealed an elevated leukocyte count of 13.6 × 10^9^/L, a very low platelet count of 24 × 10^9^/L, elevated inflammatory markers including a C-reactive protein of 399 mg/L (reference range (RR) < 5 mg/L) and a procalcitonin level of 34 ng/L (RR < 0.1 ng/L), and an elevated creatinine of 1.27 mg/dl. Her hemoglobin was only slightly reduced with 11.5 g/dl. As the patient did not present during the influenza season and first occurence of the influenza-like symptoms was parallel with the positive blood cultures, testing was not performed. Testing for HIV was negative and fasting glucose was normal. As the patient had no increased rate of infections in the past, we did not test for complement of IgG deficiency. Multiple blood cultures and swabs of the postoperative wound and the vagina were obtained and sumitted for culture.

A computed tomography scan (CT) of the chest showed diffuse bilateral alveolar infiltrates and nodular opacities with cavity forming consistent with necrotising pneumonia (Figure 1).

Transthoracal echocardiography on the day after admission showed no abnormal findings, in particular no vegetations suggestive of infective endocarditis. Community-onset pneumonia and severe sepsis with coagulopathy was diagnosed.

Empirical intravenous antibiotic therapy with piperacillin/tazobactam and levofloxacin was started immediately. On day 2, the initial abscess fluid yielded *S. aureus* fully susceptible to antistaphylococcal agents with the exception of penicillin and tetracyclin. Therefore, piperacillin/tazobactam was changed to high-dose intravenous flucloxacillin 4 g tid and levofloxacin was discontinued. On day 3, blood cultures and both wound and vaginal swab taken on admission also grew *S. aureus.* On day 4, rifampicin 600 mg Rifampicin 600 mg (once daily) was added as blood cultures continued to be positive and the patient still suffered from high fever and severe dyspnoea. CT scans of the chest and abdomen showed newly emerged extensive bilateral pleural effusions. In addition, multiple abscesses had evolved in the pectoralis, supraspinatus, and gluteus muscle. A reevaluation of the heart valves by transesophagal echocardiography revealed no abnormalities. On day 6, polymerase chain reaction (PCR) amplification of the *lukS-lukF* genes was performed as described previously [5] and confirmed the presence of the PVL gene in all available *S. aureus* isolates. Subsequently, clindamyin 600 mg tid was added on day 7. With this treatment, blood cultures became negative on day 8. On day 11, bilateral pleural drainage tubes had to be inserted as effusions increased. Cultures of the pleural fluid showed no growth. All *S. aureus* isolates from the abscess, the postoperative wound and multiple blood cultures had identical susceptibility profiles. Minimal inhibitory concentrations (MICs) were <0.25 mg/l for oxacillin, <0.25 mg/l for clindamycin, and <0.5 mg/l for rifampicin. Pulsed-field gel electrophoresis (PFGE) of *Sma*I digests of genomic DNA from all available *S. aureus* isolates showed identical patterns (data not shown).

The patient recovered very slowly with a 3 weeks stay at the intensive care unit with maximal supportive care. Respiratory support and vasopressors had not been necessary. Subsequently, the patient stayed for another 8 weeks on a regular ward until recovery. Antibiotics were continued for a total treatment duration of 5 weeks for rifampicin and clindamycin and of 8 weeks for flucloxacillin.

Swabs of persons in close contact to the patient were taken but were negative for PVL-producing *S. aureus.*

*S. aureus* is a major cause of respiratory, skin, bone, joint, and endovascular infections. Mostly these infections occur in persons with known risk factors such as cardiovascular disease, malignancy, or diabetes mellitus. *S. aureus* is responsible for at least 10% of cases of nosocomial pneumonia but only for 2% of community-acquired pneumonia [6, 7].

Our patient was a young immunocompetent woman with no apparent risk factors who sustained severe community-onset necrotizing pneumonia, multiple abscesses, and extensive pleural effusions. These clinical features are characteristic for invasive infections with *S. aureus* exhibiting the putative virulence factor PVL.

The true incidence of PVL-associated pneumonia is unknown, since the number of cases published is likely to be an underestimate. Besides its occurence in methicillin-sensitive *S. aureus* (MSSA), PVL is more often identified in community-acquired methicillin-resistant *S. aureus* (MRSA) [8].

PVL is a very virulent toxin expressed by *S. aureus*, which was first characterized in 1932. Less than 5% of all *S. aureus* strains harbor PVL genes. PVL is a pore-forming toxin destroying the membrane of the host defence cells and erythrocytes [9]. Lina et al. [5] screened *S. aureus* isolates to correlate toxin production with disease manifestation. They found a definite association between the occurrence of PVL genes and furunculosis and community-onset pneumonia. Gillet et al. [4] compared the clinical features of PVL-positive pneumonia with PVL-negative pneumonia and found significant differences. PVL-positive patients were younger without risk factors for infection. They presented more often with haemoptysis, high fever, tachycardia, tachypnoea and developed diffuse bilateral infiltrates and pleural effusion. The mortality rate was significantly higher with 75% in PVL-positive compared to 47% in PVL-negative infections. Other case series confirm the characteristics and severeness of the PVL-positive infections [10–14]. The symptoms of our patient fitted very well into this disease.

Since PVL-positive *S. aureus* strains may spread between persons in close contact to the index patient [15], swabs should be taken to prevent further spreading. In a report from Germany, control of a furunculosis outbreak involving PVL-positive MSSA in a rural village was achieved by stringent decolonization of carriers [16], but established public health recommendations do not exist.

For the therapy of necrotizing pneumonia caused by PVL-positive *S. aureus* no specific guidelines have been published. Combination therapy is widely used empirically in life-threatening infections. Bactericidal antibiotics such as *β*-lactam antibiotics are preferrd over bacteriostatic agents. For pneumonia, good penetration into the tissue has also to be considered. We added levofloxacin empirically to piperacillin/tazobactam to treat potential atypical bacteria. As soon as cultures became positive for *S. aureus* the antibiotic regimen was changed from piperacillin/tazobactam to high-dose flucloxacillin, as this is the antibiotic of choice for *β*-lactamase-positive *S. aureus.* As blood cultures remained continuously positive and the patient's condition worsened, rifampicin, another very potent antistaphylococcal antibiotic, was added. The efficacy of rifampicin as an adjunctive drug in patients with life-threatening infections remains controversial. As soon as the *S. aureus* isolates turned out to be PVL-positive, clindamycin was added. Several authors recommend the addition of clindamycin to the treatment of toxin-producing grampositive bacteria as it targets the bacterial ribosomes thereby potentially blocking the toxin production [17, 18]. New in vitro data showed an augmentation of PVL toxin production by *β*-lactam antibiotics, but no in vivo data have supported these observations so far [19, 20]. Other in vitro data showed that the addition of rifampicin or clindamycin to oxacillin inhibits PVL production [21]. PVL-positive MRSA infection requires treatment with vancomycin as the first-line agent. In severe *S. aureus* infections intravenous antibiotic therapy should be given for at least 4 weeks after the last blood culture positive for *S. aureus* . Although PVL plays a key role in the pathogenesis of necrotising pneumonia and Labandeira-Rey et al. could show in a mouse model that the PVL toxin alone might be sufficient to cause pneumonia, it is not likely to be the only virulence determinant responsible for this syndrome [2, 22].

The outcome of patients with necrotizing pneumonia may be poor in many cases even if appropriate antibiotics are administered. In addition, intensive supportive care is crucial for improving the outcome in these severe infections [23].

In our patient, necrotizing pneumonia developed shortly after incision of a Bartholin's abscess. Infection of the Bartholin's gland is the most common infectious vulvar disease and develops in approximately 2% of all women [24]. Surgical intervention is considered the primary treatment, and controversy on the benefit of antibiotics exists [25, 26]. Complication rates are very low, but cases of severe infections have been reported [27–36]. *S. aureus* appears to be only very rarely involved in these cases. Clinical complications have included septic shock, toxic-like-syndrome, necrotizing fasciitis, myocarditis, and gangrene but pneumonia has not been reported.

Bartholin's abscesses mostly contain a mixed flora of aerobic and predominantly anaerobic bacteria. The most frequently isolated aerobe is *Escherichia coli*, while *S. aureus* is only rarely detected [37, 38].

PVL-positive *S. aureus* infection should early be included in the differential diagnosis when young immunocompetent persons develop necrotizing pneumonia. Various minor infections—such as Bartholin's abscess—can precede this life threatening syndrome. Antibiotic treatment with bactericidal antistaphylococcal agents is recommended, and invasive surgical procedures may become necessary.

## Figures and Tables

**Figure 1:**
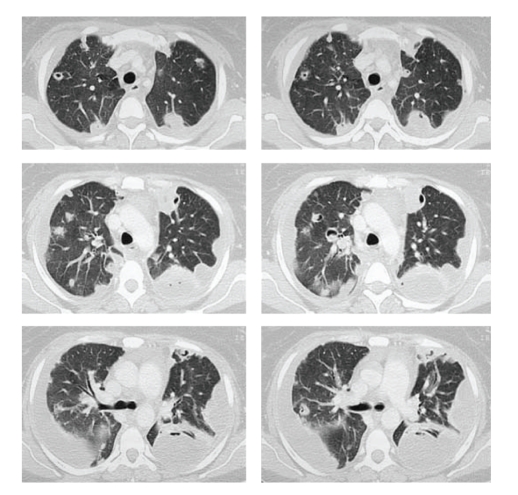
CT scan revealed nodular opacities and diffuse bilateral infiltrates.
